# Parietal and intravascular innate mechanisms of vascular inflammation

**DOI:** 10.1186/s13075-015-0528-2

**Published:** 2015-01-28

**Authors:** Giuseppe A Ramirez, Patrizia Rovere-Querini, Maria Grazia Sabbadini, Angelo A Manfredi

**Affiliations:** IRCCS Ospedale San Raffaele, via Olgettina 60, 20132 Milan, Italy; Università Vita Salute San Raffaele, via Olgettina 58, 20132 Milan, Italy

## Abstract

Sustained inflammation of the vessel walls occurs in a large number of systemic diseases (ranging from atherosclerosis to systemic vasculitides, thrombotic microangiopathies and connective tissue diseases), which are ultimately characterized by ischemia and end-organ failure. Cellular and humoral innate immunity contribute to a common pathogenic background and comprise several potential targets for therapeutic intervention. Here we discuss some recent advances in the effector and regulatory action of neutrophils and in the outcome of their interaction with circulating platelets. In parallel, we discuss novel insights into the role of humoral innate immunity in vascular inflammation. All these topics are discussed in light of potential clinical and therapeutic implications in the near future.

## The clinical spectrum of vascular inflammation

Blood vessels act as tissue integrators by granting the diffusion of oxygen, nutrients and particulate signals throughout the body. The immune function emerges during evolution as a tool to defend the circulatory system from threats to its integrity. Each static player (that is, the vessel walls) or dynamic player (that is, blood components) of the circulatory system rapidly shifts towards a defensive, inflamed state and cooperates with evolutionary more recent adaptive immune responses. Vessels might thus represent the archetypical scenario for the very early initiation of the inflammatory response.

Under physiological conditions, self-limiting inflammatory processes occur in the circulating blood that necessarily involve the vessel walls, when the immune system effectively copes with microbial and nonmicrobial threats, eliminating the original noxa and guiding vessel regeneration and eventual healing. Threats that cannot be removed or persistent deregulated immune responses directed against endogenous vascular constituents in turn underlie vascular diseases.

Atherosclerosis and its complications represent the leading cause of mortality in westernized countries and the most frequent clinical manifestations of the effects of persisting vessel inflammation. The priming event in vascular inflammation in atherosclerosis is exquisitely metabolic, since the origin of the disease is associated with the accumulation of lipoproteins endowed with oxidative potential in the intimal layer with ensuing lipidogenic persistent inflammation. The characteristic atherosclerotic lesion (that is, the atheromasic plaque) typically develops assuming an eccentric shape.

In addition to these consolidated data, novel evidence is progressively emerging about the implications of persistent vascular inflammation for a large number of systemic diseases; in particular, those diseases in which autoimmunity plays a crucial role such as systemic sclerosis (SSc), systemic lupus erythematosus (SLE), dermatomyositis and other connective tissue diseases, thrombotic microangiopathies (TMAs) and systemic vasculitides. Some of these diseases have received more significant attention in recent years and could serve as clinical and pathophysiological paradigms.

SSc is an autoimmune disease of unknown etiology, characterized by widespread organ dysfunction, peripheral ischemia and fibrotic substitution. Vascular immune-mediated injury of small arteries and capillaries is an early event in the natural history of the disease and often takes place before fibrosis is established. Endothelial activation and apoptosis are thought to constitute the priming process in the progression of vascular injury. Recent studies provide evidence for a role of neutrophil-dependent interleukin (IL)-6 signaling in mediating the early phase of vascular injury in SSc [1]. Vessel remodeling and intimal proliferation in turn could arise as a response to endothelial dysfunction and rheologic disturbances [2,3]. Endothelial cells and myofibroblasts could both be involved in neointima formation in SSc. The latter cell subset can derive from resident pericytes, transdifferentiating cells or bone marrow-derived precursors [4]. Lung involvement comprises interstitial lung disease and pulmonary arterial hypertension and represents a major issue in the management of SSc, given the high mortality rate and the poor efficacy of available treatments. Conventional immunosuppressive treatments are only partially or not at all effective in controlling and reversing vascular events (for example, pulmonary arterial hypertension) whose pathogenesis is only partially defined [2].

TMAs such as thrombotic thrombocytopenic purpura, hemolytic uremic syndrome (HUS) and pre-eclampsia are characterized by widespread endothelial injury and expression of thrombogenic stimuli such as von Willebrand factor (vWF), due to the release of endotheliotropic toxins (characteristic of typical HUS), impaired inhibition of the complement system (atypical HUS) or other noncharacterized stimuli, possibly in the setting of jeopardized ADAMTS-13 activity (thrombotic thrombocytopenic purpura) [5]. Endothelial injury/activation in turn reflects on platelets and the coagulation system, with microvascular thrombosis and end-organ ischemia.

Systemic vasculitides comprise heterogeneous diseases, characterized by persistent inflammatory damage of the vessel walls [6]. According to the Chapel Hill Consensus Conference, there are seven classes of systemic vasculitides: large vessel vasculitides (including giant cell arteritis (GCA) and Takayasu’s arteritis), small vessel vasculitides (including anti-neutrophil cytoplasmic antibody-associated vasculitides (AAV), IgA vasculitis and cryoglobulinemic vasculitis), medium vessel vasculitides (including Kawasaki’s disease and polyarteritis nodosa), variable vessel vasculitides (including Behçet’s disease), single organ vasculitides, vasculitides associated with systemic disease and vasculitides associated with probable etiology. Although the pathogenetic mechanisms and clinical scenarios differ, the diseases share the inflammatory involvement of vessels as the primary event in the disease natural history and the associated multiorgan systemic involvement.

## Blood vessel checkpoints: role of vessel-residing cells in the initiation of the inflammatory response

**Figure 1:**
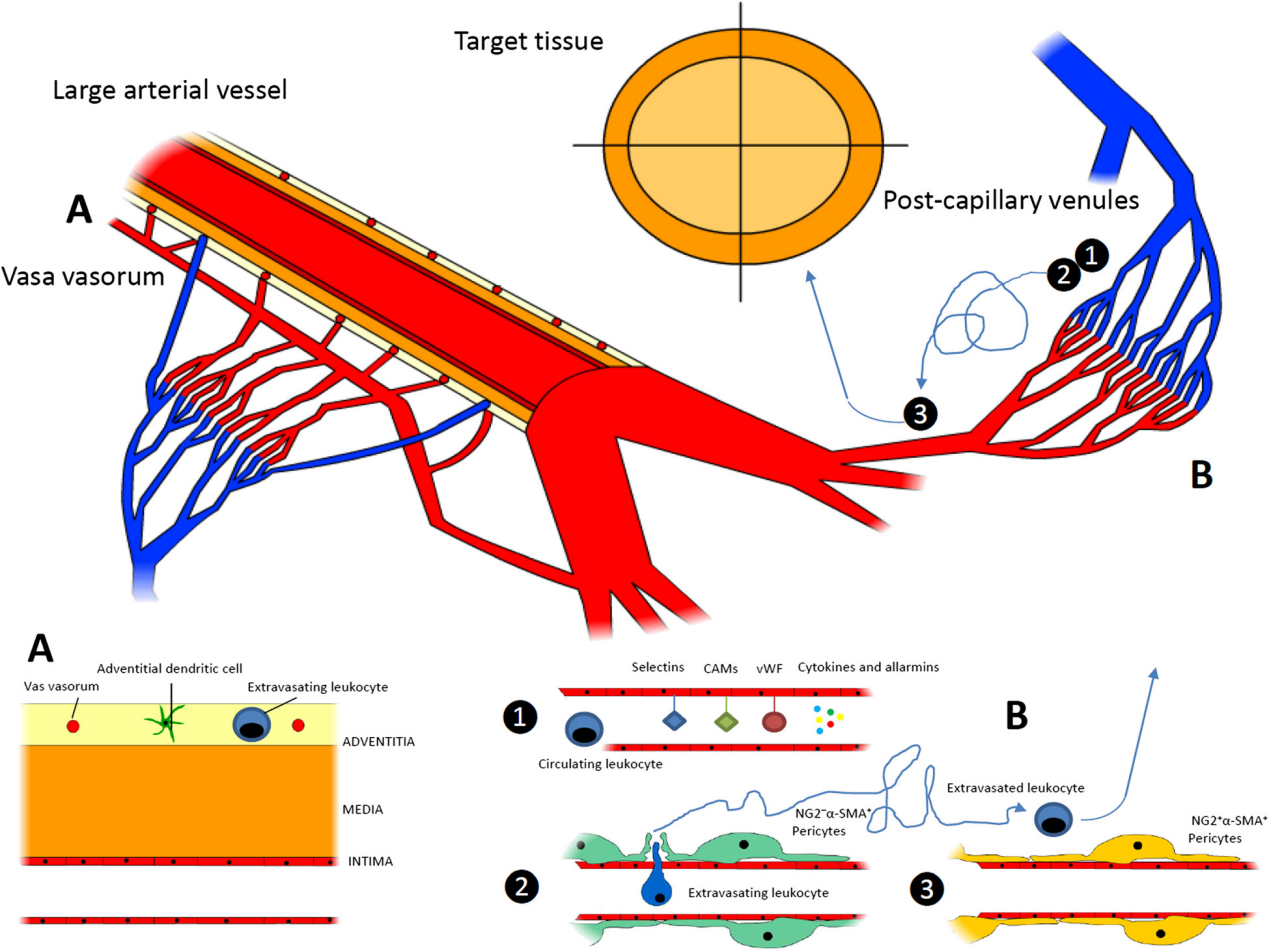
**Checkpoints on leukocyte migration through inflamed vessels and tissues.** In the setting of inflamed large arterial vessels, leukocytes adhere to the walls of the *vasa vasorum* and eventually extravasate through them. After accessing the adventitial layer of large arteries, leukocytes interact with vessel-residing dendritic cells that induce the generation of a follicle-like structure during chronic inflammation **(A)**. In general, circulating leukocytes that approach an inflamed tissue **(B)** interact with signaling molecules expressed by activated endothelial cells (and platelets), which promote their adhesion to the vessel walls and eventually their migration through endothelial cells and perivascular connective tissue **(1)**. This process mainly occurs at the level of postcapillary venules, where leukocytes are assisted by a first subset of neuron–glial antigen 2-negative/alpha-smooth muscle actin-positive (NG2^−^ α-SMA^+^) pericytes **(2)**. Both luminal and pericyte-derived signals enhance leukocyte survival and activation. As an example, neutrophils exposed to vascular cell adhesion molecule 1 (VCAM1) live longer: notably VCAM1 is required for the full development of anti-neutrophil cytoplasmic antibody-associated vasculitides glomerulonephritis, while its soluble and endothelial surface expression in rheumatoid arthritis correlates with joint damage and late-stage vascular injury, respectively. After diapedesis, leukocytes move through the interstitial space, mainly following slow and nonlinear routes. Interactions with a second subset of NG2^+^α-SMA^+^ arteriolar/capillary pericytes prompt leukocytes to progress faster and more linearly towards target tissues and might be involved in perpetuating vascular inflammation **(3)**. CAM, cell adhesion molecules; vWF, von Willebrand factor.

Circulating leukocytes interact with cells that resides within the vessel walls as well as with other circulating cells that interact with blood vessels in order to gain information about ongoing damage in surrounding tissues and eventually to extravasate. To this purpose, either cells located in the lumen of blood vessels or cells located at the periphery of the vessel wall are able to productively interact with circulating and extravasating leukocytes and drive their subsequent effector responses. Cells that define the internal wall of blood vessels, such as the endothelium or the platelets recruited at sites of vessel injury to surrogate the function of the endothelium, indeed sense potential threats to the integrity of vessels and surrounding tissues through an array of pattern recognition receptors (PRR) [7,8]. After rapid mobilization of intracellular stores, endothelial cells and platelets expose a large array of signaling molecules such as P-selectin (from the endothelial and platelet side), vWF (from endothelial Weibel–Palade bodies) and inflammatory signals such as the high mobility group box 1 protein, the soluble form of CD40 ligand, leukotrienes LTA4 and LTB4 and tissue factor [7]. Later responses involve the transcriptionally regulated synthesis of E-selectin, vascular cell adhesion molecule 1 and intercellular adhesion molecules (Figure 1B1).

Rolling/crawling neutrophils, monocytes and innate-like lymphocytes, besides being directly activated by the original inflammatory stimuli, recognize the changes on the vessel cell surface, and in turn generate signals that expand the sensitivity of blood vessels [6]. The recognition of potentially harmful agents in the context of blood vessels thus prompts the development of a tripartite crosstalk involving the endothelium, platelets and leukocytes [7].

During migration towards target tissues, leukocytes (at least of the myeloid lineage) receive additional information after interacting with vascular pericytes (Figure 1B2,B3). These poorly defined cells, wrapped around the endothelium, play a role that has only recently gained attention as a secondary pre-tissue checkpoint. Venular neuron–glial antigen 2-negative/alpha-smooth muscle actin-positive (NG2^−^ α-SMA^+^) pericytes directly provide navigation support during extravasation, modifying their shape in response to inflammatory signals forming gaps and thus providing preferential exit routes to neutrophils through the venular wall [9] (Figure 1B2). By contrast, capillary or arteriolar neuron–glial antigen 2-positive/alpha-smooth muscle actin-positive (NG2^+^ α-SMA^+^) pericytes recruit myeloid leukocytes after completion of diapedesis (even from relatively distant sites) and enhance their survival as well as the speed and the linearity of their migration in the perivascular interstitial space (Figure 1B3). As such, this latter pericyte subset might be specifically involved in the maintenance of inflammation associated with small arteries and capillaries [10]. Pericytes contribute to the remodeling of vessels and of surrounding tissues under conditions of hypoxia [4,11] and regulate the vascular tone, possibly by acquiring vascular smooth muscle cell-like features [11].

Spreading of inflammation through large arterial vessel walls involves unique pathophysiological pathways. In fact, large arteries are themselves vascularized, as they are served by a specific set of small vessels in the adventitial layer, called the *vasa vasorum*. Adventitial dendritic cells are thought to coordinate the recruitment of activated CD4^+^ T lymphocytes from the *vasa vasorum* and to influence the deployment of T-helper (Th)1/Th17-driven immune responses in the underlying vessel layers [12] (Figure 1A). Interactions between vessel-residing dendritic cells and T cells have been extensively studied in GCA and are required for full establishment of the disease [13]. However, evidence that similar phenomena also occur in atherosclerosis is progressively being acquired [14]. In the specific setting of GCA, two cytokine clusters apparently drive the inflammatory process: (a) the steroid-sensible IL-6/IL-17 cluster, which would be sustained by Th17 cells; and (b) the IL-12/interferon gamma cluster due to the persistent activity of Th1 cells, responsible for the progression or refractoriness of the disease despite corticosteroid or anti-IL-6 drugs [13].

Downstream of the activation of vessel-residing dendritic cells and extravasated lymphocytes, stromal cells are thought to participate in vessel inflammation by providing quantitative and qualitative alterations of the extracellular matrix. (1) Quantitative expansion of the extracellular matrix, together with the proliferation of stromal cells and infiltrating leukocytes, is responsible for vessel thickening and eventually occlusion. (2) In addition, the disruption of the histological architecture of the vessel wall is accompanied by changes in the signal that the extracellular space provides to residing and infiltrating cells. In particular, recent evidence suggests that imbalances in the immune-regulatory functions of the stromal microenvironment (for example, signaling through galectin 1A, fibronectin and syndecan) could be responsible for the constitutional activation of dendritic cells in large vessel vasculitides [13]. Endothelial dysfunction constitutes one of the pathogenic hallmarks of atherosclerosis, which is also characterized by a centrifugal development of vessel inflammation from the luminal side towards the medial and adventitial layers. By contrast, large vessel vasculitides are thought to be characterized by a centripetal pattern of inflammation. Nonetheless, signs of endothelial activation are also detectable in patients with GCA [15,16] and recent studies support a role of anti-endothelial antibodies in causing vascular injury in this context [17].

## Innate players in vascular inflammation

### Neutrophil effector functions

Myeloid cells and neutrophils in particular undergo an acute burst of activation during very early phases of atherothrombosis [18] and play a pivotal role in the initiation and perpetuation of vascular and tissue inflammation in small and medium vessel vasculitides [19], SLE [20], SSc [1,21] and other inflammatory illnesses. During acute inflammatory responses, regardless of whether in response to microbial or sterile inciting stimuli, these cells are recruited from the blood to sites of inflammation. After recognition of endothelial-derived, platelet-derived and eventually pericyte-derived signals, activated neutrophils: (a) firmly adhere to the vessel walls and eventually extravasate, while granule contents (myeloperoxidase (MPO), proteinase 3 (PR3), the targets of anti-neutrophil cytoplasmic antibodies in AAV – and pentraxin 3 (PTX3) in particular; see below) migrate to the cell surface and are eventually released; (b) undergo an oxidative burst and generate reactive oxygen species; (c) become resistant to programmed cell death and as such prolong their survival; and (d) in certain conditions generate neutrophil extracellular traps (NETs) [22] – that is, extracellular grids of decondensed and extensively modified (for example, by citrullination) chromatin that enhance the host response to pathogens by providing locally high concentrations of microbicidal moieties and by promoting immunothrombosis [23].

**Figure 2:**
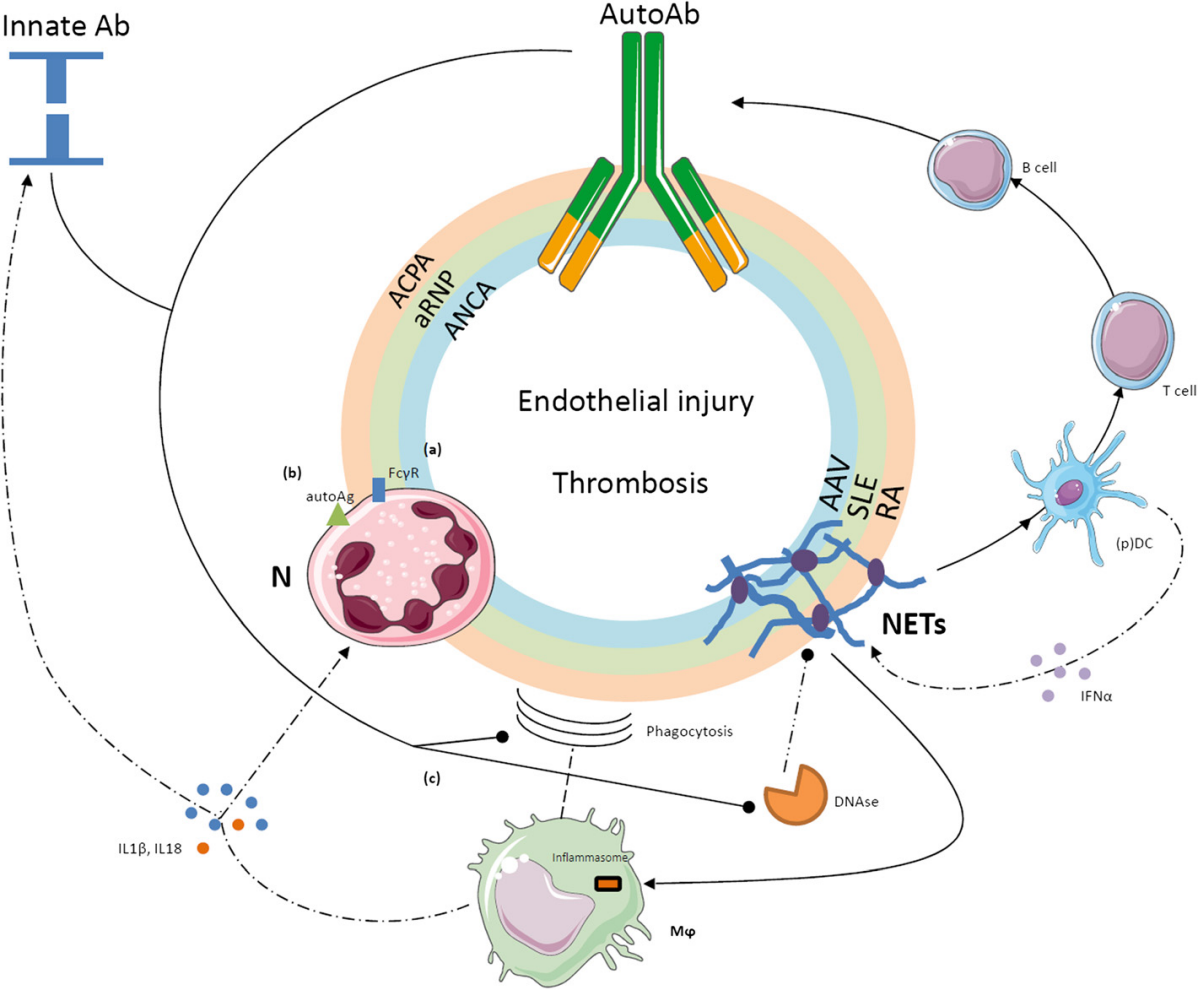
**Self-sustaining and amplifying feedback loops of NETosis and subsequent humoral response.** Enhanced neutrophil activation and the accumulation of neutrophil extracellular traps (NETs) characterize several autoimmune diseases. NETs are a source of diverse antigens that include myeloperoxidase, proteinase 3, human neutrophil peptide (HNP), the chemotactic peptide LL37, ribonucleoproteins, citrullinated residues, various nuclear proteins as well as DNA itself. Owing to the adjuvant effect of NET-associated signals such as the high mobility group box 1 protein, autoantigens are productively processed by dendritic cells (DC) and presented to T cells that undergo productive activation and favor the production of autoantibodies upon clonal expansion, proliferation and differentiation of antigen-specific B lymphocytes. Recognition of nucleic acids by plasmacytoid dendritic cells (pDC) induces the release of interferon alpha (IFNα), which in turn promotes NET generation. Autoantibodies generated against neutrophil nuclear or cytosolic components such as anti-citrullinated peptides (ACPA), anti-ribonuclear protein (aRNP) or anti-neutrophil cytoplasmic antibody (ANCA) antibodies as well as anti-LL37, anti-HNP, anti-DNA and anti-DNAse antibodies promote neutrophil activation and NETosis: **(a)** by direct interaction with immunoglobulin receptors (FcγR) on the cell membrane, **(b)** by recognizing their target antigens on the surface of neutrophils and **(c)** through inhibition of the clearance of NET by macrophages and hindrance with their enzymatic degradation by enzymes such as DNAse. Locally produced soluble pattern recognition receptors, such as pentraxin (PTX)3, could be implicated at various levels. On the other hand, NET-derived nuclear components recognized by macrophage promote the assembly of inflammasomes with eventual extensive release of cytokines such as interleukin (IL)-1β and IL-18. These in turn promote neutrophil activation and the synthesis of innate humoral mediators such as PTX3, which can further affect NET clearance. AAV, ANCA-associated vasculitides; Ab, antibody; Ag, antigen; N, neutrophil; Mϕ, macrophage; RA, rheumatoid arthritis; SLE, systemic lupus erythematosus.

Neutrophils also interact directly with adaptive immunity by recruiting Th1 and Th17 at sites of inflammation and by supporting B-cell survival and maturation [23,24]. Furthermore, neutrophils influence vessel permeability and might influence the regulation of the coagulation cascade by prompting thrombosis, an event that has been proposed recently to exert a protective action by ensnaring intravascular microbes (immunothrombosis). Neutrophils themselves constitute a circulating source of autoantigens and inflammatory signals, which become exposed to the immune system during neutrophil activation and/or NETosis and might in certain conditions contribute to autoimmunity [24,25]. In physiology, NETs contribute to the host response to invading microbes and, when generated at high neutrophil density, aggregate and favor the termination of the inflammatory response by degrading cytokines and interfering with the further activation and recruitment of neutrophils [26]. However, neutrophils also contribute to the establishment of a vicious circle sustaining inflammation in SLE [27-29], rheumatoid arthritis (RA) [30] and AAV [31] (Figure 2).

NETting neutrophils expose an alarming burden of autoantigens such as DNA, citrullinated histones, MPO and PR3 [28,30,31]. Upon cross-presentation, they prompt the generation of autoantibodies recognizing DNA (anti-DNA), RNP (anti-ribonuclear protein (aRNP)), LL37 peptide, citrullinated moieties (anti-citrullinated peptides (ACPA)) as well as MPO and PR3 (anti-neutrophil cytoplasmic antibody (ANCA)) [28,30,31]. Antibodies in turn promote neutrophil activation, NETosis and persistence of NETs through: (a) the direct engagement of surface molecules, such as self-antigens like LL37 peptide, MPO and PR3 [32], and immunoglobulin receptors (FcγR); or (b) the impairment of regulation mechanisms such as enzymes involved in the degradation of NETs (for example, DNAse) [27] or interference with the function of cells involved in the phagocytic clearance of neutrophil antigens (that is, macrophages). Under physiological conditions, NET clearance is indeed a relatively uneventful process, which shares with the clearance of early apoptotic cells the lack of production of inflammatory signals, such as IL-1β, IL-6 and tumor necrosis factor (TNF) [33]. Disturbance in the process could conversely result in amplification of bystander inflammatory events [33].

The events initiating the loop are poorly understood. An immature inflammatory phenotype has been shown to characterize circulating neutrophils in SLE [20,34]. This particular subset of low-density granulocytes is characterized by an enhanced bactericidal signature that results in an increased tendency to NETosis. NETting low-density granulocytes induced endothelial injury *in vitro* and were associated with the development of vasculitis in patients with SLE [20]. Neutrophils from RA patients easily undergo NETosis and in this process generate a panel of circulating moieties of possible diagnostic interest [35]. In a similar way, neutrophils from patients with SSc showed an enhanced response to inflammatory stimuli even in the absence of priming [21]. On the other hand, patients with AAV and RA have enhanced, genetically determined expression of PR3 on the surface of neutrophils [36]. Enhanced spontaneous, platelet-induced or antibody-induced neutrophil activation, possibly through NETosis, results in a fallout of potentially dangerous events, such as endothelial injury and thrombosis [37].

In particular, immunothrombosis is emerging as a novel intravascular effector of innate immunity [25]. In contrast to hemostasis, immunothrombosis occurs in intact blood vessels, results from the activation of multiple innate processes involved in antimicrobial responses and could thus cooperate with other neutrophil effector functions, such as NETosis, in the development of sepsis [22]. NETosis plays a fundamental role in promoting thrombosis since NETs neutralize regulatory agents of the coagulation cascade such as tissue factor pathway inhibitor and activating factor XII, and employ captured vWF and histone proteins to enhance platelet recruitment and activation [25,34]. On the other hand, the expression of intravascular tissue factor by activated platelets and immune cells contributes to the development of immunothrombosis through the activation of the coagulation cascade [25]. The activation of thrombin can in turn potentiate the activation of the endothelium and of circulating platelets through protease-activated receptors [7].

### Neutrophil anti-inflammatory responses

Myeloid-derived suppressor cells comprise heterogeneous immature myeloid cells with immunosuppressive properties, arising from the bone marrow when systemic perturbation in the cytokine network occurs during cancer or inflammation [24]. It could be attractive to speculate about a possible application of these cells in a setting of adoptive immuno(suppressive) therapy. However, the milieu required for generating myeloid-derived suppressor cells is poorly characterized [38]. Pillay and colleagues have recently identified a specific CD16^bright^CD62L^dim^CD11b^bright^CD11c^bright^ neutrophil subset, elicited by systemic inflammation, that showed reduced expression of adhesion molecules and impaired extravasation and suppressed T-cell proliferation [38]. Suppressor neutrophils are detectable in patients with GCA and their systemic expansion is apparently modulated by steroid therapy. Moreover, they could be induced *ex vivo* by high concentrations of IL-17 and IL-6 [39]. The source of the functional instruction required to promote the differentiation of suppressor neutrophils has not yet been identified, and the nature of their involvement in the natural history of large vessel vasculitis, whether causative or epiphenomenal, remains to be defined.

### Platelets and their interactions with leukocytes

**Figure 3:**
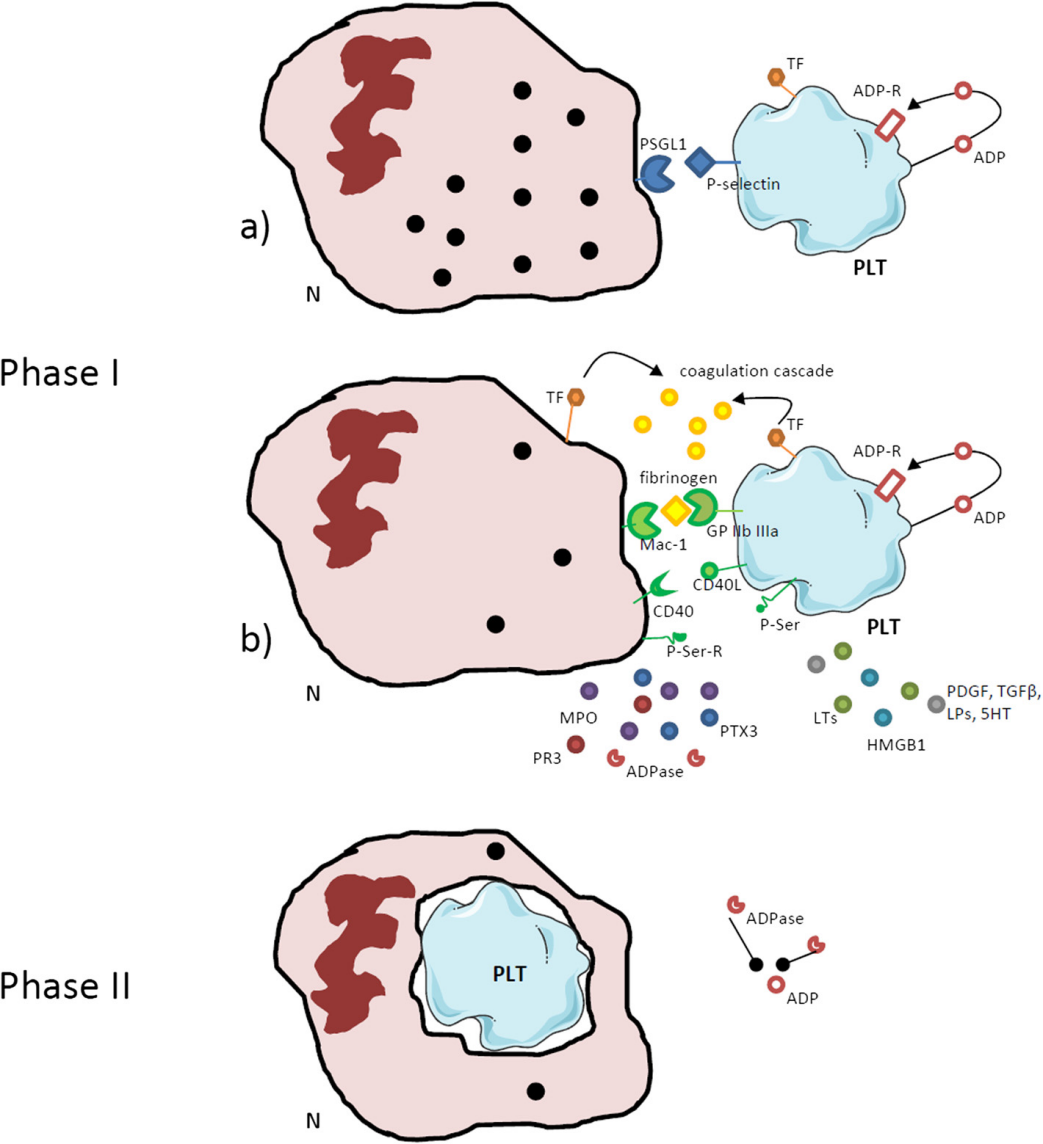
**Platelet–leukocyte interactions. (Phase I)** Neutrophils (N) interact with circulating platelets (PLT): after recognition of platelet P-selectin by the P-selectin granulocyte ligand 1 (PSGL1), neutrophils implement their engagement with platelets by upregulating Mac-1 (also known as α_M_β_2_ or CD11b/CD18), a surface integrin that interacts with platelet-bound fibrinogen in cooperation with glycoprotein (GP) IIbIIIa (also known as α_2b_β_3_ integrin). The activation of neutrophils (possibly further enhanced by CD40–CD40 ligand (CD40L) interactions) results in the release of enzymatic moieties such as myeloperoxidase (MPO), proteinase 3 (PR3) and of the prestored soluble pattern recognition receptor pentraxin (PTX)3 as well as in the expression of tissue factor (TF), which in turn promotes thrombin generation. Platelets also release various bioactive signals such as leukotrienes (LTs), high mobility group box 1 protein (HMGB1), platelet-derived growth factor (PDGF), transforming growth factor beta (TGFβ), lysosphingolipids (LPs), and 5-hydroxytryptamine (5HT). **(Phase II)** Recognition of phosphatidylserine (P-Ser) on platelets prompts their phagocytic clearance and quenches their procoagulant capacity; neutrophil ADPases break the auto/paracrine loop of ADP-mediated platelet activation. Neutrophils that had phagocytosed platelets become largely anergic after degranulation.

Enhanced platelet activation and deregulated interactions with circulating leukocytes occur in the setting of small vessel vasculitides [40] and large vessel vasculitides [7], as well as in SSc [41], transfusion-related acute lung injury [37,42] and other inflammatory conditions. Platelet–leukocyte aggregation reflects interactions physiologically occurring after acute vessel injury: during this process, neutrophils undergo an early burst of activation, characterized by degranulation and tissue factor expression. Extensive adhesion culminates in platelet phagocytosis and eventual neutrophil anergy (Figure 3) [41]. Platelet–leukocyte heterotypic aggregates occur in several diseases in which vessel involvement is prominent, ranging from atherothrombosis [43] to vasculitis [7]. The outcomes of platelet–leukocyte cross-talk widely differ and are thought to significantly affect the global ischemic risk. In particular, a failure in the homeostatic control of the reciprocal platelet and neutrophil activation could acutely cause thrombosis, associated with accelerated neutrophil extravasation and bystander vessel/tissue damage. Chronically, it might jeopardize vessel and/or perivascular tissue healing [3]. Significant efforts have been spent in recent years on the quest for safer and more efficacious anti-platelet agents. Besides an undisputed protective action in patients with cardiovascular and thromboembolic diseases, some agents might exert additional effects on vascular inflammation that could be valuable in the context of systemic vasculitis and SSc (Table 1) [44].

**Table 1:** Possible therapeutic impact of selected anti-platelet agents in systemic vasculitides and systemic sclerosis

**Drug**	**Mechanism of action**	**Possible applications**
Aspirin	• COX inhibition	• Inhibition of vessel remodeling in large vessel vasculitides (conflicting results in clinical trials)
	○ TXA2 inhibition	○ Inhibition of vessel remodeling and vasoconstriction (possibly in synergy with PGI2 analogues) in SSc (limited data supporting modest efficacy in clinical settings)
	○ Inhibition of EGFR signaling and reduction of VEGF, MMP and IL-12 production	
	• Non-COX-dependent inhibition of leukocyte infiltration of the vessel walls	
Dipyridamole	• Adenosine reuptake inhibition and cyclic nucleotide phosphodiesterase inhibition	• Inhibition of PDGF-mediated vessel remodeling and platelet-leukocyte interactions in giant cell arteritis
	• Interference with platelet–leukocyte aggregation	• Vasodilation and interference with vessel and tissue remodeling in SSc (promising results from preclinical studies, but nonconclusive results in clinical studies)
	• Inhibition of PDGF secretion from platelets	
Vorapaxar, atopaxar	• Thrombin receptor (PAR-1) antagonism	• Prevention of thrombotic events due to enhanced endothelial and platelet activation in vasculitides with frequent thrombosis (for example, Buerger’s disease, Behçet’s disease or AAV)
	• Inhibition of platelet activation	
	• Inhibition of endothelial activation	
Sarpogrelate	• Serotinin 5HT_2A,B_ receptor antagonism: impaired loading and release of serotonin by platelets	• Inhibition of fibrosis and vessel remodeling and reduced pulmonary hypertension in SSc (two small Japanese studies report apparent benefits in the control of skin ulcers and improvement in right ventricular ejection fraction, CO diffusion and pulmonary arterial pressure)
	○ Inhibition of serotonin-induced vasoconstriction	• Inhibition of vessel remodeling and pulmonary hypertension in Behcet’s disease, Takayasu’s arteritis and other inflammatory conditions
	○ Inhibition of serotonin-mediated endothelial toxicity	
	○ Inhibition of serotonin-induced platelet activation	
	○ Inhibition of serotonin-induced fibrosis	

AAV, anti-neutrophil cytoplasmic antibody-associated vasculitides; CO, carbon monoxide; COX, cyclooxygenase, EGFR, epidermal growth factor receptor; IL, interleukin; MMP, matrix metalloproteinases; PAR-1, protease activated receptor-1; PDGF, platelet-derived growth factor; SSc, systemic sclerosis; TXA2, thromboxane A2; VEGF, vascular endothelial growth factor.

### The complement system: an old dog learning new tricks

**Figure 4:**
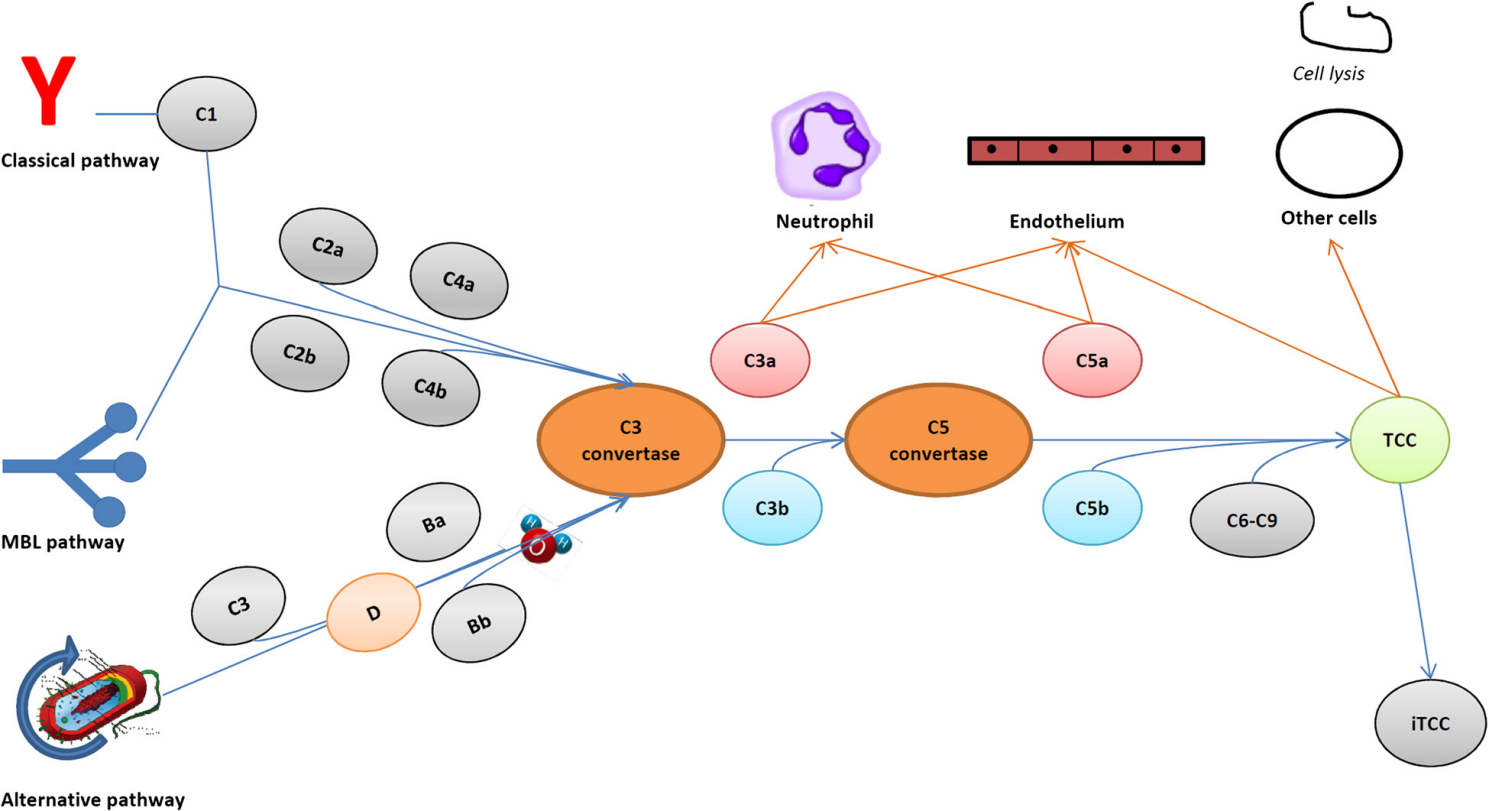
**The complement system. Effectors:** Irrespective of the activation pathway, three families of molecules are generated during the complement cascade: complement opsonins (C3b and C4b), complement anaphylotoxins (C3a and C5a) and the terminal complement complex (TCC). The key common step in the progression of the complement cascade is the generation of a C3-convertase, which cleaves inactive C3 into C3a and C3b. The latter binds to the C3-convertase to generate a C5-convertase, which generates C5a and C5b from inactive C5. C5b interacts with factors 6 to 9 to establish the TCC, which induces cell lysis by acting as a membrane attack complex or accumulates in the fluid phase or in extravascular spaces as an inactive moiety (iTCC). It can also bind to cell membranes as a sublytic membrane attack complex. **Activators:** Both in the classical and the lectin pathway, cleaving enzymes (namely component C1r and C1s of the C1 factor in the classical pathway and mannose binding lectin (MBL)-associated serine proteases in the MBL pathway) are destabilized and activated by antigen–antibody interactions (either directly in the case of MBL or with the intermediation of component C1q in the classical pathway) to process C4 to C4a, C4b and C2a, C2b respectively. The C4bC2a complex corresponds to the first variant of C3-convertase. Moieties expressed on the bacterial surface determine in the alternative pathway the spontaneous generation of the C3bBb complex, which acts as the second solid phase variant of C3-convertase. This atypical form of C3-convertase develops when partial spontaneous activation of C3 (tickover) is accompanied by binding of C3 with factor B, which in turn is cleaved to factor Ba and Bb by factor D to generate the C3(H_2_O)Bb complex or fluid-phase C3-convertase.

The complement system comprises an arsenal of plasma proteins sequentially activated by diverse stimuli to converge towards the generation of opsonins, anaphylotoxins and a terminal complement complex with prominent cytolytic functions [45] (Figure 4). Activation of the complement system, of the endothelium and of neutrophils and platelets is intermingled *in vivo* [45]. Recognition of complement metabolites (such as the anaphylotoxins C3a and C5a or of noncytolytic forms of terminal complement complex) by the endothelial layer enforces a feed-forward loop, with enhanced surface expression of adhesion molecules, tissue factor and vWF [45], which in turn favors the activation of blood neutrophils and platelets. The generation of anaphylotoxins directly impacts on neutrophil activation, while platelet activation facilitates the further activation of the complement cascade, which in turn amplifies thrombin-dependent platelet aggregation. Humoral immunity triggers complement activation and genetically determined or acquired immune defects influence the risk of developing vascular inflammation (Table 2) [45,46]. On the other hand, surface molecules (for example, CD46, CD55, CD59) and soluble molecules (for example, factor H, factor I, vitronectin, clusterin) that quench the complement activation play a crucial role in the protection of vessels and of the perivascular tissues [45].

**Table 2:** **Humoral innate response and complement in vascular inflammation**

**Antibody**	**Activation pathway**	**Pathogenic role**	**References**
CRP	Classical	• Binds to oxidized lipoproteins or apoptotic cells in atherosclerosis together with terminal complement complex	[61]
PTX3	Classical alternative	• Supposed protective role in atherosclerosis	[16,59,60,62,63]
		• Highly expressed in inflamed vessels of small and large vessel vasculitis (function unknown)	
MBL	MBL alternative	• Excess or defect in serum levels associate with increased risk of intimal hyperplasia and ischemic cardiopathy in RA, Kawasaki disease and in the general population	[53,54,64-68]
		• Implication in the activation of complement and in microbial clearance in IgA vasculitis	
		• Marginal role in GCA, AAV and Behçet’s disease	

AAV, anti-neutrophil cytoplasmic antibody-associated vasculitides; CRP, C-reactive protein; GCA, giant cell arteritis; MBL, mannose binding lectin; PTX, pentraxin; RA, rheumatoid arthritis.

Defective control and/or enhanced activation of the complement system are key pathogenic factors in TMAs [5,47]. TMA-like features are also detectable in other autoimmune diseases such as SSc, small vessel vasculitis, SLE and anti-phospholipid syndrome, which possibly share a deregulated activation of the alternative pathway of complement activation [48]. Intravascular assembly of terminal complement complex is detectable in the early phases of atherosclerosis and precedes monocyte infiltration and foam cell formation [45], while the deficiency of C3 is associated with larger atherosclerotic lesions [49]. Activation of the complement system in immune-complex-associated small vessels vasculitis results in endothelial damage by means of cytolysis and as a consequence of the recruitment of neutrophils through anaphylotoxins [50]. Also in AAV, which has been traditionally thought of as ‘pauci-immune’ complement activation, the complement system is required for priming neutrophils to express PR3 on the cell surface and plays an increasingly appreciated role in the disease’s natural history [50]. NETs and NETs bound to autoantibodies activate complement, which in turn impairs NET degradation, a feature of major pathogenic and therapeutic interest in SLE and AAV [29]. The role of complement in large vessel vasculitides to date is controversial.

Anti-complement agents are being developed and tested. Current strategies comprise enhanced complement inhibition (for example, by means of recombinant Ig/complement regulator fusion proteins), direct inhibition of C3 activation (soluble CR1 or anti-C3 antibodies), blockade of anaphylotoxins receptors or inhibition of the common terminal cascade (that is, mainly C5 inhibition) [51]. Intravenous immunoglobulins constitute the first anti-complement agent [51]. They proved efficacious in a variety of clinical settings including severe or refractory middle-size or small vessel vasculitides [6]. Eculizumab is an anti-C5 monoclonal antibody currently used for atypical HUS with promising potential applications in other TMA settings and perhaps in acute coronary syndromes. An open-label trial is currently being performed to test the efficacy of eculizumab in patients with a history of catastrophic anti-phospholipid syndrome undergoing renal transplantation [NIH:NCT01029587]. By contrast, a phase II trial [NIH:NCT01275287] in AAV was withdrawn due to failure in participant recruitment. Another phase II trial is currently recruiting participants to test the efficacy of CCX168, a C5a receptor antagonist, in addition to cyclophosphamide in AAV [NIH:NCT01363388].

### Collectins, pentraxins and other soluble pattern recognition receptors

Humoral innate immunity consists of invariant molecules (soluble PRR) such as pentraxins, collectins and ficolins that, during the early phases of the inflammatory response, dispose of autoantigens and discriminate between noxious and harmless stimuli [46,52]. PRR are functionally correlated to immunoglobulins, since they also show opsonic, neutralizing and complement-activating functions [46]. Some PRR are produced on demand and are systemically active (for example, C-reactive protein (CRP)), whereas others are constitutively produced (for example, serum amyloid protein P) or only locally produced (for example, PTX3) [46]. Furthermore, innate PRR can either favor or dampen the progression of inflammation and tissue injury, depending on the nature of the initiating stimuli (for example, necrotic vs apoptotic cells), the pre-existing inflammatory context (for example, septic vs sterile inflammation) and the expression profile of inhibitory or proinflammatory receptors on target cells [46]. It is often difficult deciphering the net effect of PRR in the pathogenesis of inflammatory conditions, including those characterized by vessel inflammation. The interaction of PRR with the complement cascade plays a striking role in modulating vessel susceptibility to inflammation and injury (Table 2).

#### Collectins and ficolins

Collectins are PRR characterized both by the ability to recognize carbohydrate patterns (lectins) and by an evolutionary link with collagen molecules. Mannose binding lectin (MBL) is one of the most representative members of this class of PRR, as it constitutes the prototypic trigger of the MBL pathway for the activation of complement. Serum levels of MBL were quadratically related to carotid intima-media thickness in patients with RA [53]. Similar quadratic functions could perhaps be employed to describe the relationship between serum levels of MBL and the risk of coronary lesions in Kawasaki’s disease and IgA vasculitis [54] (Table 2). In the setting of Kawasaki’s disease it has been recognized that higher MBL expression correlates with cardiac disease in patients of older age. On the other hand, lower expression of MBL associates with enhanced risk of coronary complications in younger children, possibly because of defective protection against airborne infectious triggers [54]. MBL and class A immunoglobulins act synergically and MBL mediates the activation of complement after recognition of pathogens by polymeric IgA [55]. Consequently, MBL plays a major role in the development of IgA vasculitis and IgA-related nephritis (Berger’s disease) both as an enhancer of complement activation and as a determinant of respiratory pathogen clearing efficiency (Table 2). The impact of MBL in the pathogenesis of GCA, AAV and Behçet’s disease seems to be modest, while protection against adverse effects of infections and against complications of atherosclerosis and atherothrombosis in particular could both contribute to explain the high frequency of MBL etherozygous mutations [56].

Ficolins share many structural elements with collectins and are characterized by the presence of an N-terminal collagen-like domain involved in the activation of the MBL pathway and a C-terminal lectin domain. They comprise a membrane-bound protein (M-ficolin or ficolin 1) and two soluble ficolins (L-ficolin or ficolin 2 and H-ficolin or ficolin 3), which form in the blood complexes with MBL-associated serine proteases and with their truncated proteins, an event that upon interaction with carbohydrates exposed on the microbial surface in turn leads to proteolytic activation of the complement pathway. Ficolins are involved in the modulation of the immune response against a wide range of bacterial and fungal species. Genetic variations in the expression of ficolins could play a role in aberrant antimicrobial responses during Behçet’s disease [57].

#### Pentraxins

The pentraxin family comprises a wide number of soluble and membrane-bound PRR, which can be further subdivided into short and long pentraxins. The former group includes serum amyloid protein P and CRP, whereas PTX3, pentraxin 4, neuronal pentraxins 1 and 2 and their receptor are comprised in the latter [46].

CRP is probably the most widely used inflammatory biomarker, due to its rapid liver-centered response to inflammatory stimuli and in particular to IL-6. From a molecular point of view, the elevation of CRP levels during the acute phase provides a first-line antibody response against invading microbes: the opsonic effect of CRP is achieved through the recognition of phosphorylcholine residues on the surface of pathogens; furthermore, CRP activates the classical complement pathway when in the soluble phase [46]. In recent years, elevations of circulating CRP have also emerged as markers of metabolic disease and cardiovascular risk. A role for CRP as a facilitator of the scavenging of cellular apoptotic debris due to excessive metabolic cellular stress (Table 2) could be implicated [46], even if extreme caution should be exercised in the use of CRP as a biomarker or as an indicator of a pathogenetic inflammatory component common to diverse cardiovascular events [58].

PTX3 is a prototypic long pentraxin and a local modulator of the inflammatory response. In the acute phase, PTX3 acts as the humoral partner of the first-line neutrophil response. Upon activation, neutrophils release PTX3 from secondary granules, thus exhausting their nonrenewable preformed stores, and build up a PTX3-enriched antimicrobial environment during NETosis [46]. PTX3 also interferes with the P-selectin/PSGL1 pathway, thus counter-regulating the process of neutrophil extravasation and endothelial/platelet-assisted activation [7]. Furthermore, in contrast to CRP, PTX3 activates the classical complement pathway in the solid phase and exerts an inhibitory effect in the liquid phase [46]. Accordingly, PTX3 circulating levels rise suddenly during acute ischemia and predict mortality in myocardial infarction, sepsis and intestinal ischemia [46].

In later stages of tissue inflammation, however, constitutive production of PTX3 from non-neutrophil sources takes over and systemic blood concentrations of PTX3 correlate poorly with disease activity. In this setting, PTX3 regulates the load of autoantigens recognized by resident and infiltrating phagocytes [52] and interacts with matrix components, growth factors and other inflammatory moieties (for example, TNF-stimulated gene 6 protein) [46] to modulate tissue and vessel proliferation during inflammation [46]. Increased expression of PTX3 has been detected in atherosclerotic lesions [46]. PTX3 is also significantly expressed at sites of vessel remodeling in GCA and Takayasu’s arteritis [16,59] as well as in leukocytoclastic lesions in AAV (Table 2) [60]. Enhanced release of PTX3 from endothelial cell and myofibroblasts might reflect SSc-related persistent vessel inflammation and defective vasculogenesis [3]. On the basis of its ability to dampen tissue injury through regulation of neutrophil access to the extravascular space [7] and to quench maladaptive vessel remodeling, therapeutic applications of PTX3 in the setting of sterile vessel injury have been proposed [46].

## Conclusions

Humoral and cellular innate immunity both contribute to the origin of vessel inflammation, to its acute complications and to the long-term vascular remodeling that underlies vessel injury and end-organ ischemia. Early alterations of the barrier function of vessels, which rely on both endothelial cells and pericytes, license leukocytes for vessel wall and surrounding tissue infiltration and for ensuing acute and chronic inflammatory responses. Neutrophils and platelets are key interacting players in the initiation and perpetuation of vascular inflammation. We are acquiring insight about the role of humoral innate immunity in physiologically shaping the extent and the specific features of inflammation throughout the vascular system. These regulator mechanisms are jeopardized in persistent inflammatory diseases and might represent crucial targets for restoring vascular homeostasis.

## Abbreviations

AAV: Anti-neutrophil cytoplasmic antibody-associated vasculitides
ACPA: Anti-citrullinated peptides antibodies
ANCA: Anti-neutrophil cytoplasmic antibodies
aRNP: Anti-ribonuclear protein
CRP: C-reactive protein
GCA: Giant cell arteritis
HUS: Hemolytic uremic syndrome
IL: Interleukin
MBL: Mannose binding lectin
MPO: Myeloperoxidase
NET: Neutrophil extracellular trap
PR3: Proteinase 3
PRR: Pattern recognition receptors
PTX3: Pentraxin 3
RA: Rheumatoid arthritis
SLE: Systemic lupus erythematosus
SSc: Systemic sclerosis
TCC: Terminal complement complex
Th: T-helper
TMA: Thrombotic microangiopathy
TNF: Tumor necrosis factor
TSG-6: TNF-stimulated gene 6 protein
vWF: von Willebrand factor
